# SIRT1 regulates glycolysis and angiogenesis of ovarian cancer through β-catenin/c-Myc/PKM2, and its mechanism in chemotherapy resistance

**DOI:** 10.3389/fonc.2026.1762850

**Published:** 2026-03-04

**Authors:** Chengju Zhang, Hu Wang, Yu Zhou, Deng He, Tiantian Feng, Xi Wang, Shangqi Ni, Juan Zhang

**Affiliations:** 1 Department of Gynecology, Affiliated Hospital of North Sichuan Medical College, Nanchong, Sichuan, China; 2 North Sichuan Medical College, Nanchong, Sichuan, China

**Keywords:** angiogenesis, chemotherapy resistance, glycolysis, ovarian cancer, SIRT1, β-catenin

## Abstract

**Background:**

The purpose of this study is to explore the molecular mechanism of SIRT1 regulating chemotherapy resistance.

**Methods:**

Expression level of SIRT1 in ovarian cancer cell lines SKOV3, SKOV3/DDP (cisplatin-resistant cell line) and normal ovarian epithelial cell line IOSE80 was detected. Through the intervention of β-catenin agonist BML-284, the mechanism of SIRT1 regulating glycolysis and angiogenesis through β-catenin/c-myc/PKM2 pathway was discussed. Finally, the nude mice transplanted tumor model was constructed to verify the role of SIRT1 *in vivo*.

**Results:**

The expression of SIRT1 increased in ovarian cancer cell line, especially in cisplatin-resistant cell line SKOV3/DDP. Knocking down SIRT1 can inhibit the proliferation, invasion and migration of ovarian cancer cells, promote cell apoptosis, and reduce the drug resistance of cells to cisplatin. SIRT1 enhances the malignant biological behavior of ovarian cancer cells by promoting glycolysis and angiogenesis. SIRT1 up-regulates the expression of key glycolytic enzymes and angiogenic factors by activating β-catenin/c-myc/PKM2 pathway. *In vivo* experiments, knocking down SIRT1 can reduce the glycolysis level and angiogenesis ability of tumor tissue.

**Conclusion:**

SIRT1 promotes glycolysis and angiogenesis of ovarian cancer cells by activating β-catenin/c-myc/PKM2 pathway, thus enhancing chemotherapy resistance. SIRT1 is expected to be a new target for ovarian cancer treatment.

## Introduction

Ovarian cancer is one of the common malignant tumors in female reproductive system, and its incidence is on the rise worldwide (1). Because the early symptoms were not obvious, most patients were in the late stage when they were diagnosed, and missed the best treatment opportunity. At present, the main treatments for ovarian cancer include surgical resection, chemotherapy and radiotherapy. Among them, the platinum-based chemotherapy scheme is the first-line treatment scheme for ovarian cancer (2). However, although the initial treatment is effective, most patients will eventually develop chemotherapy resistance, leading to disease recurrence and poor prognosis. Therefore, it is very important to study the molecular mechanism of chemotherapy resistance of ovarian cancer and find new therapeutic targets for improving the survival rate of patients.

Metabolic reprogramming of tumor cells is one of the important characteristics of tumor occurrence and development. Warburg effect, also known as aerobic glycolysis, means that tumor cells still prefer glycolytic pathway to get energy under the condition of sufficient oxygen. Glycolysis not only provides energy for tumor cells, but also provides intermediate metabolites for cell growth and proliferation (3). More and more studies show that glycolysis plays an important role in the occurrence, development, metastasis and drug resistance of tumors (4). In addition, angiogenesis is a necessary condition for tumor growth and metastasis. Tumor cells secrete angiogenic factors, which promote the formation of tumor blood vessels, provide nutrition and oxygen for tumors, and promote the metastasis of tumor cells (5). Therefore, inhibiting glycolysis and angiogenesis of tumors is expected to become a new strategy for treating tumors.

SIRT1 (silent information regulator 1) is a NAD+-dependent deacetylase belonging to Sirtuin family. SIRT1 plays an important role in many physiological processes of cells, including DNA repair, apoptosis, metabolic regulation and inflammatory response (6). In recent years, it has been found that SIRT1 is abnormally expressed in many kinds of tumors, which is closely related to the occurrence and development of tumors (7). In ovarian cancer, the expression of SIRT1 is usually increased, which is associated with poor prognosis (8, 9). At the same time, previous studies have revealed that SIRT1 is closely related to the drug resistance of ovarian cancer, and chemotherapy sensitivity can be enhanced by regulating SIRT1 (8, 10–13). These studies indicate that SIRT1 may affect the chemosensitivity of ovarian cancer cells by regulating apoptosis, promoting Polyploid giant cancer cells and epithelial-mesenchymal transition. However, the specific mechanism of SIRT1 in chemotherapy resistance of ovarian cancer remains to be further studied.

The innovation of this study is that we pay attention to the influence of SIRT1 on metabolic reprogramming of ovarian cancer cells, and choose β-catenin/c-Myc/PKM2 pathway as the research focus, based on the following considerations: firstly, β-catenin is the key molecule of Wnt signaling pathway, which plays an important role in the occurrence and development of tumors. Secondly, c-Myc is an important transcription factor, which is involved in the regulation of cell proliferation, differentiation and apoptosis. PKM2 is a key enzyme in glycolytic pathway, and its increased expression can promote glycolysis of tumor cells. Previous studies have shown that β-catenin, c-Myc and PKM2 play an important role in glycolysis, angiogenesis and drug resistance of tumors (14–17). Therefore, we speculate that SIRT1 may affect the glycolysis and angiogenesis of ovarian cancer cells by regulating the β-catenin/c-Myc/PKM2 pathway, and then regulate chemotherapy resistance.

Based on the above background, this study aims to explore the molecular mechanism by which SIRT1 affects glycolysis and angiogenesis of ovarian cancer cells and regulates chemotherapy resistance. This study will provide a new theoretical basis and potential target for the treatment of ovarian cancer.

## Materials and methods

### Cell line and cell culture

Human ovarian cancer cell line SKOV3 and its cisplatin-resistant variant SKOV3/DDP were purchased from the cell bank of China Academy of Sciences. Normal human ovarian epithelial cell line IOSE80 is preserved in our laboratory. SKOV3 and SKOV3/DDP cells were cultured in DMEM medium (Gibco) containing 10% fetal bovine serum (FBS, Gibco) and 1% penicillin/streptomycin (P/S, Gibco). IOSE80 cells were cultured in DMEM medium (Gibco) containing 10% FBS and 1% p/s. All cells were cultured in an incubator with 37°C and 5% CO2.

### Main reagents and instruments

Cisplatin (CDDP): purchased from Sigma-Aldrich Company; β-catenin agonist BML-284: purchased from Tocris Bioscience company; Apatinib: purchased from Jiangsu Hengrui Pharmaceutical Co., Ltd.; SIRT1 siRNA: synthesized by GenePharma Company; Lipofectamine 3000 transfection reagent: purchased from Thermo Fisher Scientific Company; CCK8 kit: purchased from Dojindo Company; Annexin V-FITC/PI Apoptosis Kit: purchased from BD Biosciences; Matrigel matrix adhesive: purchased from Corning company; Glucose uptake test kit: purchased from Abcam Company; Lactic acid detection kit: purchased from Abcam Company; ATP test kit: purchased from Beyotime company; ROS detection kit: purchased from Beyotime company; NADPH/NADP+ detection kit: purchased from Beyotime Company; Western Blotting related antibodies: antibodies such as SIRT1, β-catenin, c-myc, PKM2, HK2, LDHA, GLUT1, VEGFA, Ang-1, IL-8 and GAPDH were purchased from Cell Signaling Technology, Abcam and Santa Cruz Biotechnology Company; QRT-PCR related reagents: RNA extraction kit, reverse transcription kit and SYBR Green qPCR Mix were purchased from Takara Company; Flow cytometry: BD FACSCanto II; Enzyme marker: Bio-Rad iMark; Inverted microscope: Olympus IX71.

### Cell grouping

In this study, cells were divided into the following groups according to the experimental purpose:

(1)Ctrl group: Cells were not treated.
(2)Si-NC group: low expression negative control group.
(3)Si-SIRT1 group: SIRT1 cells were under-expressed.
(4)Si-NC- BML-284 group: low expression negative control+β-catenin agonist BML-284(10 mM).
(5)Si-SIRT1-BML-284 group: SIRT1-knockdown cells treated with β-catenin agonist BML-284 at 10 mM.

### SiRNA transfection

SKOV3 and SKOV3/DDP cells were inoculated into a 6-well plate at a density of 5×105 cells per well, and cultured until the cell density reached 70-80%. SIRT1 siRNA was transfected into cells according to the instructions of Lipofectamine 3000 transfection reagent. After transfection for 6 hours, the fresh medium was replaced, and the follow-up experiment was carried out after 48 hours of continuous culture.

### Western blot experiment

Protein was extracted from cells or tissues by RIPI lysis buffer to obtain total protein samples. SDS-PAGE electrophoresis: the concentration of protein was determined by BCA protein determination method, and electrophoresis was carried out with 10% and 12% polyacrylamide gel (SDS-PAGE), and the loading mass was 40 μ g per well; After electrophoresis at 80 V for 120 min, the protein was transferred to polyvinylidene fluoride (PVDF) membrane at 300 mA and 90 min, and sealed with 5% skim milk powder at room temperature for 1 h;; The PVDF membrane was washed with PBST for 4 times (5 min/time), and the target protein band was cut according to the position of Marker, and put into the incubator. Add the target proteins SIRT1, HXK2, PKM, LDHA, PDHA1), c-mycALDOA, GLUT1, VEGFA, Ang-1, IL-8, β-catenin, TCF1/TCF7, MMP-7, PKM2 and mouse-derived GAPDH respectively, and then wash PVDF membrane 4 with PBST. Accord/ming to the source of antibody species, the goat anti-rabbit or mouse polyclonal antibody (1:10000, China Kangwei Reagent Biotechnology Co., Ltd.) combined with horseradish peroxidase was added, and incubated for 1 h; at room temperature in a shaking table at the speed of 70 ~ 80 rpm. The PVDF film was washed with PBST for 4 times (5 min/time), and the signal of the combination of the secondary antibody and the primary antibody was detected by ECL color development. The photo was taken by chemiluminescence detection system, and the ratio of the optical density of the target strip to GAPDH was detected by Image J. Collect cells or tumor tissues, add RIPA lysate (containing protease inhibitor) and crack on ice for 30 minutes. Centrifuge at 4°C and 12000rpm for 15 minutes, and collect supernatant. The protein concentration was determined by BCA method. Take the same amount of protein for SDS-PAGE electrophoresis, and then transfer the protein to PVDF membrane. Sealed with 5% skim milk for 1 hour, and then added with primary antibody (incubated overnight at 4°C). TBST washes the membrane three times, each time for 10 minutes. Add secondary antibody (incubate at room temperature for 1 hour). TBST washes the membrane three times, each time for 10 minutes. ECL development and Image J software were used to analyze the gray value of protein bands.

### qRT-PCR

Total RNA was extracted from samples (cells, tissues, etc.) with Trizol reagent, and the total RNA was quantified with Qubit fluorometer. The extracted RNA was reverse transcribed into cDNA, and 2g of total RNA was taken and reverse transcribed using a high-capacity cDNA reverse transcription kit (Applied Biosystems, USA). The reaction procedures were 25°C for 5 min, 50°C for 15 min and 85°C for 5 s;Prepare a PCR reaction system, put the reverse transcribed cDNA and primers into a PCR reaction tube, and carry out PCR reaction with ABI-7300 fluorescence quantitative PCR instrument. The reaction conditions are denaturation at 95°C for 10 s, annealing at 60°C for 20 s, extension at 72°C for 15 s, and repeat 40 cycles. The relative expression level of each RNA is determined by QCPR using SYBR GreenMaster reagent (American Applied Biological Systems Company). The gene and primer sequences are shown in **Table 1**. The Llevels of genes were have been quantified using the 2-ΔΔCT method., and the result was expressed as mean standard deviation.

**Table 1:** Primer sequence.

Gene	Sequences
SIRT1	F: TGACCTCCTCATTGTTATTGGG R: GGCATACTCGCCACCTAACCT
HK2	F: GCCAGAGCATCCTCCTCAAGTG R: TCACCACAG CAACCACATCCAG
PKM2	F:ATTATTGAGGAAC TCCGCCGCCT R:ATTCCGGGTCACAGCAA TGATGG
LDHA	F:ATGGCAACTCTAAAGGATCA R:GCAACTTGCAGTTCGGGC
PDHA1	F:ATGGAATGGGAACGTCTGTTG R:CCTCTCGGACGCACAGGATA
ALDOA	F:GGTGAGGAGGAGGAGGAGG R:CCTGCTGCTGCTGCTGCTG
GLUT1	F: GTGGATTGAGGGTAGGAGGTTTGG R: GAGCAAGAGGACACTGATGAGAGG
VEGFA	F:GGTGAGGAGGAGGAGGAGG R:CCTGCTGCTGCTGCTGCTG
Ang-1	F:GGTCTTCATACTGGGTCTGGGTCTG R:TCGTAGTGCTGGGTCAGGAGTG
IL-8	F:ACTGAGAGTGATTGAGAGTGGAC R:AACCCTCTGCACCCAGTTTTC
β-catenin	F:CTTCCAGACACGCCATCATG R:CACACAGAGTACTTGCGCTC
TCF1	F:GGTGAGGAGGAGGAGGAGG R:CCTGCTGCTGCTGCTGCTG
MMP-7	F:CATGATTGGCTTTGCGCGAG R:AGACTGCTACCATCCGTCCA
C-myc	F:GGCTCCTGGCAAAAGGTCA R:CTGCGTAGTTGTGCTGATGT
GAPDH	F:TGCAACCGGGAAGGAAATGA R:GCATCACCCGGAGGAGAAAT

### CCK8 experiment

Cells were inoculated into 96-well plates at a density of 2×103 cells/well, cultured for 24 hours, and then treated with different concentrations of cisplatin (0, 2.5, 5, 10, 20 μM). After 48 hours of continuous culture, 10 μl CCK8 reagent was added to each well and incubated for 2 hours. The absorbance value was detected at 450 nm by enzyme-labeled instrument.

### Cell clone formation experiment

Take logarithmic growth cells, blow them with trypsin, suspend them in 1640 medium containing 10% FBS and double antibody, and incubate them in an incubator for 2 to 3 weeks. Discard the supernatant and wash it carefully with PBS twice. Cells were fixed with 4% paraformaldehyde in 5mL for 15 minutes. Then remove the stationary solution, add an appropriate amount of Giemsa for 10 ~ 30 min, and then slowly rinse the dyeing solution with running water to calculate the clone formation rate.

### Cell scratch test

First, mark the back of the 6-hole plate with a marker pen, compare it with a ruler, and draw a horizontal line evenly, about every 0.5~1cm, and cross the hole. Each hole passes through at least 5 lines, and about 5 X 105 cells are added in the air; The next day, compare the gun head with the ruler, and try to hang down to the horizontal scratches behind. The gun head should be vertical and not inclined; Wash the cells with PBS for three times, remove the scratched cells, add serum-free medium, and put them in an incubator for culture. Take samples and take photos at 0,24 hours.

### Cell Transwell experiment

Take out the matrix adhesive from -20°C and melt it in the refrigerator at 4°C overnight. Then, the matrix glue was diluted with serum-free medium according to a certain proportion and evenly coated on the upper layer of Transwell chamber; Add the cell suspension to the upper layer of Transwell chamber, and pay attention to control the number and volume of cells. Put the Transwell chamber into a 24-well plate, and add the lower culture medium to put the 24-well plate into a cell incubator for culture according to the experimental requirements; After the culture, the Transwell chamber was taken out, washed with PBS, and then cell fixation and staining were carried out. The cell invasion was observed under the microscope, and the cell count was carried out.

### Flow cytometry

Cell samples of each group were collected, and the cells were washed twice with precooled PBS to remove the interference in the culture medium. Then, the cells were resuspended with Annexin V binding buffer, and the cell concentration was adjusted to an appropriate range. Then, according to the proportion of the kit instructions, respectively add Annexin V-FITC and PI dye solution, gently mix well, and incubate at room temperature in the dark for 15–30 minutes. Finally, add an appropriate amount of Annexin V binding buffer to stop the reaction, and use flow cytometry to detect as soon as possible (usually within 1 hour).

### Glucose uptake and lactic acid determination

According to the instructions of the kit, cells were labeled with fluorescently labeled deoxyglucose analog 2-NBDG, and glucose uptake was evaluated for 30 minutes. Subsequently, the cells were obtained by flow cytometry and the data were analyzed. For lactic acid determination, lactic acid concentration in whole cell lysate was detected by lactic acid determination kit according to the instructions.

### Determination of ATP, ROS and NADPH/NADP+

According to the instructions of the kit, the ATP level, ROS level and NADPH/NADP+ ratio of the cells were detected respectively.

### Tube forming experiment

Matrigel matrix glue was spread in a 96-well plate and incubated at 37°C for 30 minutes. Cells were seeded on Matrigel at a density of 2×104 cells/well. After 24 hours of culture, the tube formation was observed.

### Animal experiment

BALB/c female nude mice aged 4–6 weeks were selected and purchased from Beijing Weitong Lihua Experimental Animal Technology Co., Ltd. All animal experiments are approved by the Ethics Committee. SKOV3/DDP cells were resuspended with PBS, and the cell concentration was adjusted to 1×108 cells/ml. 100 μl cell suspension was injected subcutaneously into each mouse. On the second day after tumor implantation, mice were randomly divided into the following 7 groups (6 mice in each group):

Si-NC+CDDP group: SKOV3/DDP cells transfected with si-NC were intraperitoneally injected with normal saline.
Si-NC+BML-284+CDDP group: SKOV3/DDP cells transfected with si-NC were intraperitoneally injected with BML-284(20 mg/kg).
Si-SIRT1+CDDP group: SKOV3/DDP cells transfected with si-SIRT1 were intraperitoneally injected with normal saline.
Si-SIRT1+Apatinib+CDDP group: SKOV3/DDP cells transfected with si-SIRT1 were intraperitoneally injected with apatinib (50 mg/kg).
Si-SIRT1+BML-284+CDDP group: SKOV3/DDP cells transfected with si-SIRT1 were intraperitoneally injected with BML-284(20 mg/kg).

The other two groups were not treated with cisplatin:

Si-NC group: low expression negative control.
Si-SIRT1 group: SIRT1 cells were low-expressed.

The tumor volume was measured once a day by three experimenters independently or showed signs of ulcer. The tumor size in current work is between 474.37-1579.67 mm^3^. Then, the mice were killed, the tumor tissue was taken out and weighed. Euthanasia procedures were performed in strict accordance with the guidelines of the Institutional Animal Care and Use Committee (IACUC) and followed the AVMA Guidelines for the Euthanasia of Animals (2020 edition). Fora anesthesia, mice were first anesthetized with 3-5% isoflurane in an induction chamber with oxygen flow rate of 1 L/min, anesthesia was maintained with 1.5-2% isoflurane via nose cone. Depth of anesthesia was confirmed by loss of righting reflex, absence of toe pinch response and stable respiratory rate. Euthanasia was performed by carbon dioxide asphyxiation, briefly, carbon dioxide have been filled at 20-30% chamber volume replacement per minute, followed by cervical dislocation as secondary physical method. Some tumor tissues were used for Western Blotting and qRT-PCR analysis, and some tumor tissues were used for immunofluorescence staining. The blood of mice was collected and the indexes of liver and kidney function were detected. This study is reported in accordance with ARRIVE guidelines, and the animal study was approved by ethical committee of Affiliated Hospital of North Sichuan Medical College hospital.

### Immunofluorescence staining

The tumor tissue was embedded in paraffin and sliced. Dewaxing, antigen repair. Blocked with 5% BSA for 1 hour. Add primary antibodies (SIRT1 and CD31) and incubate overnight at 4°C. Add secondary antibody and incubate at room temperature for 1 hour. DAPI staining. Observe and take photos with a fluorescent microscope.

### Statistical analysis

In this study, all the experiments were repeated biologically for three times (n=3). Each experiment is carried out independently to ensure the reliability and repeatability of the results. In addition, in each experiment, the key experimental steps (such as cell proliferation, migration and invasion experiments) were also repeated to verify the consistency of the experimental results. All experimental data were statistically analyzed by SPSS 22.0 software. The measurement data is expressed as mean standard deviation (SD). T test was used for comparison between the two groups, and one-way analysis of variance (ANOVA) was used for comparison between multiple groups, and the difference was statistically significant (*P* < 0.05).

## Results

### SIRT1 expression increased in ovarian cancer cells

**Figure 1:**
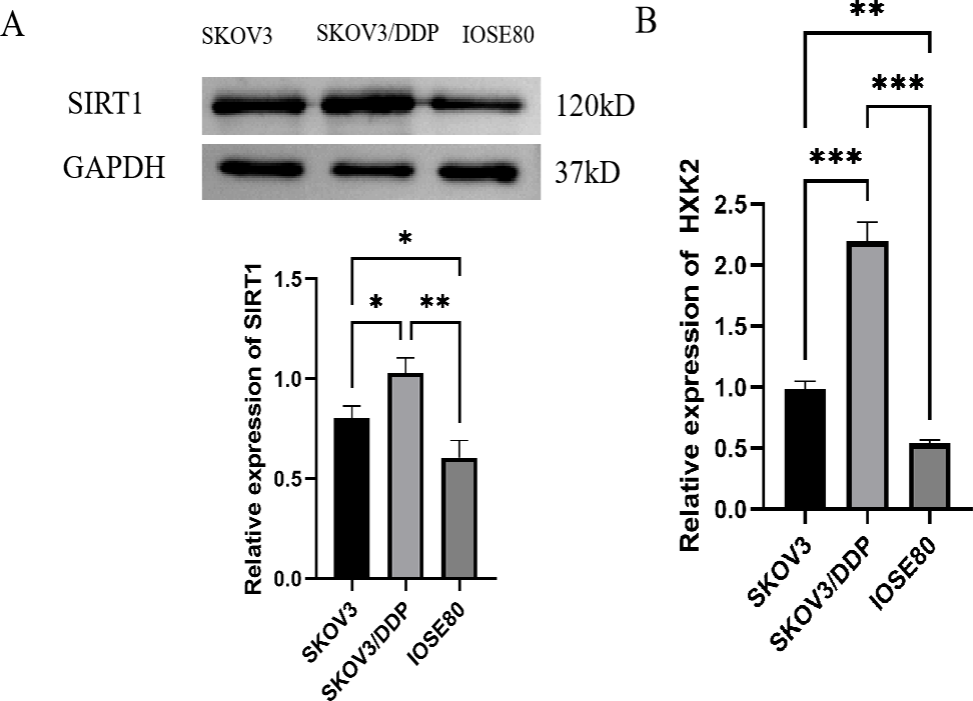
The expression of sirt1 is increased in ovarian cancer cells. **(A)** Western Blotting was used to detect the protein expression level of SIRT1 in SKOV3, SKOV3/DDP and IOSE80 cells. **(B)** qRT-PCR was used to detect the mRNA expression level of SIRT1 in SKOV3, SKOV3/DDP and IOSE80 cells. *P < 0.05, **P < 0.01, ***P < 0.001.

In order to explore the role of SIRT1 in ovarian cancer, we first detected the expression level of SIRT1 in ovarian cancer cell lines SKOV3, SKOV3/DDP and normal ovarian epithelial cell line IOSE80. Western Blotting results showed that the protein expression level of SIRT1 in SKOV3 and SKOV3/DDP cells was significantly higher than that in IOSE80 cells (**Figure 1A**). The results of qRT-PCR also showed that the mRNA expression level of SIRT1 in SKOV3 and SKOV3/DDP cells was significantly higher than that in IOSE80 cells (**Figure 1B**). In addition, the expression level of SIRT1 in cisplatin-resistant cell line SKOV3/DDP is higher than that in SKOV3 cells, suggesting that SIRT1 may be related to chemotherapy resistance of ovarian cancer.

### Knocking down SIRT1 inhibits the proliferation, invasion and migration of ovarian cancer cells and promotes apoptosis

**Figure 2:**
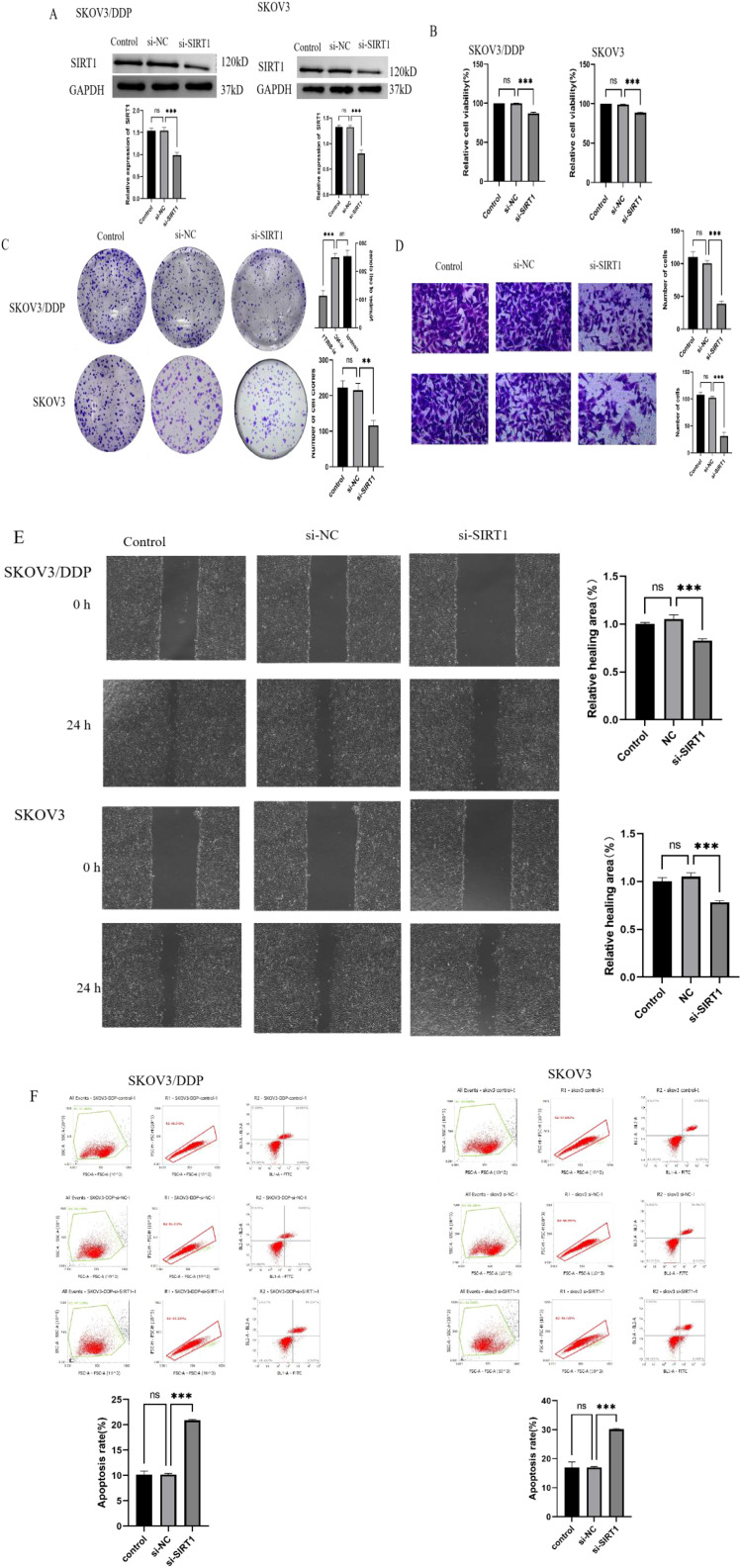
Knocking down SIRT1 inhibits the proliferation, invasion and migration of ovarian cancer cells and promotes apoptosis. **(A)** Western Blotting was used to detect the expression level of SIRT1 protein in si-NC group and si-SIRT1 group. **(B)** CCK8 test was used to detect the proliferation ability of cells in si-NC group and si-SIRT1 group. **(C)** Clonal formation experiment was used to detect the clonal formation ability of cells in si-NC group and si-SIRT1 group. **(D)** Transwell experiment was used to detect the invasion ability of si-NC group and si-SIRT1 group. **(E)** Scratch test was used to detect the migration ability of cells in si-NC group and si-SIRT1 group. **(F)** The apoptosis rate of si-NC group and si-SIRT1 group was detected by flow cytometry. **P < 0.01, ***P < 0.001.

In order to study the effect of SIRT1 on the biological behavior of ovarian cancer cells, we constructed SKOV3 and SKOV3/DDP cell lines with low expression of SIRT1. Western Blotting results showed that the expression level of SIRT1 protein in si-SIRT1 group was significantly lower than that in si-NC group (**Figure 2A**), which indicated that SIRT1 low-expression cell line was successfully constructed. The results of CCK8 experiment showed that knocking down SIRT1 could significantly inhibit the proliferation of SKOV3 and SKOV3/DDP cells (**Figure 2B**). The results of clone formation experiments show that knocking down SIRT1 can significantly reduce the number of clone formation of SKOV3 and SKOV3/DDP cells (**Figure 2C**). Transwell experiment and scratch experiment show that knocking down SIRT1 can significantly inhibit the invasion and migration ability of SKOV3 and SKOV3/DDP cells (**Figures 2D, E**). The results of flow cytometry showed that knocking down SIRT1 could significantly promote the apoptosis of SKOV3 and SKOV3/DDP cells (**Figure 2F**). This suggests that knocking down SIRT1 can inhibit the biological malignant behavior of ovarian cancer cells.

### Knocking down SIRT1 inhibits cisplatin resistance by regulating glycolysis and angiogenesis of ovarian cancer cells

**Figure 3:**
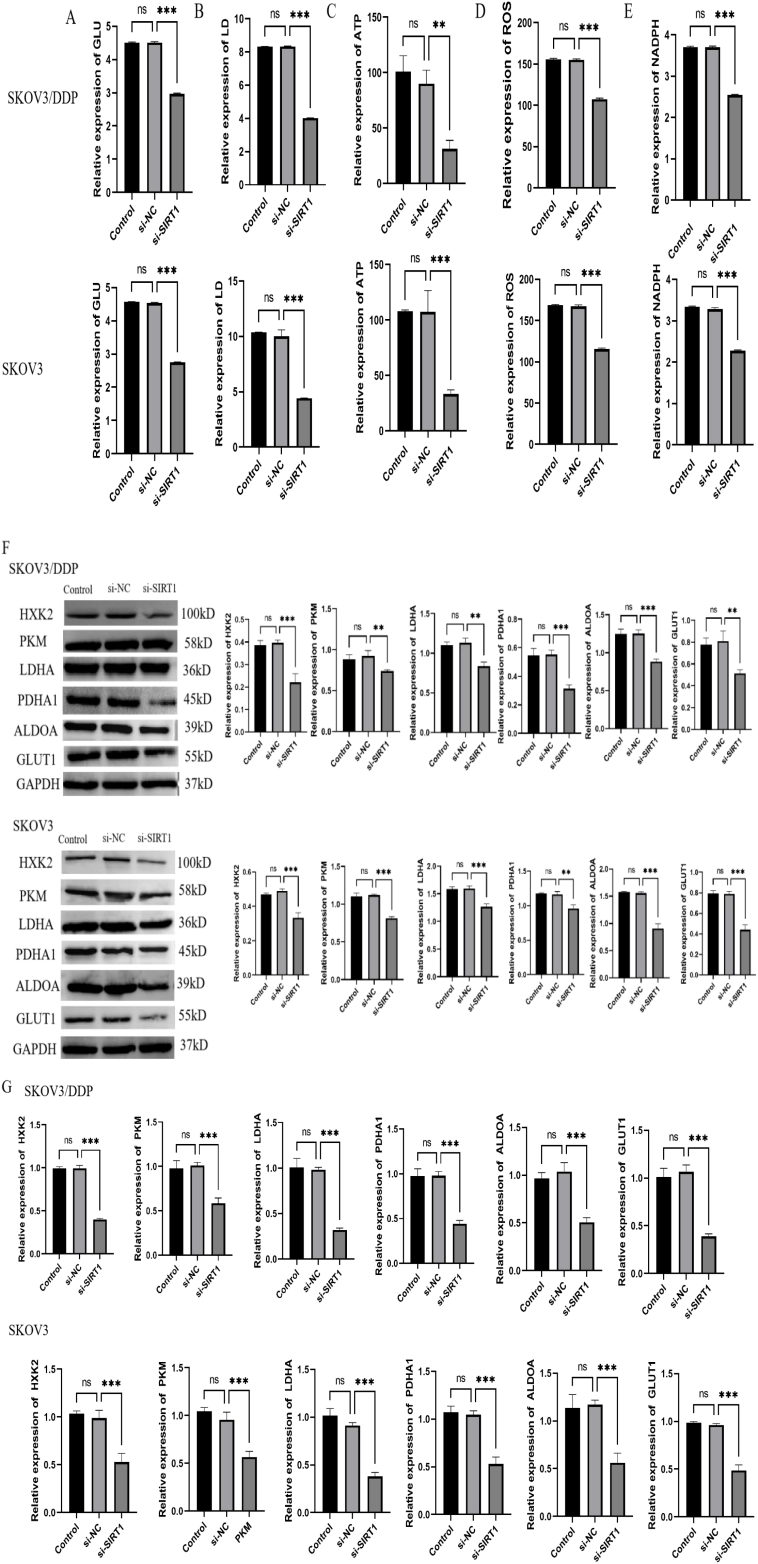
Suppression of glycolysis of ovarian cancer cells by knocking down SIRT1. **(A)** The glucose uptake ability of cells in si-NC group and si-SIRT1 group was detected by glucose uptake test. **(B)** The lactic acid production of cells in si-NC group and si-SIRT1 group was detected by lactic acid determination experiment. **(C)** ATP level of cells in si-NC group and si-SIRT1 group was detected by ATP determination experiment. **(D)** ROS level of cells in si-NC group and si-SIRT1 group was detected by ROS determination experiment. **(E)** NADPH/NADP+ ratio of cells in si-NC group and si-SIRT1 group was detected by NADPH/NADP+ determination experiment. **(F)** Western Blotting was used to detect the protein expression levels of key glycolytic enzymes (HK2, PKM2, LDHA, GLUT1) in si-NC group and si-SIRT1 group. **(G)** qRT-PCR was used to detect the mRNA expression levels of key glycolytic enzymes (HK2, PKM2, LDHA, GLUT1) in si-NC group and si-SIRT1 group. **P < 0.01, ***P < 0.001.

**Figure 4:**
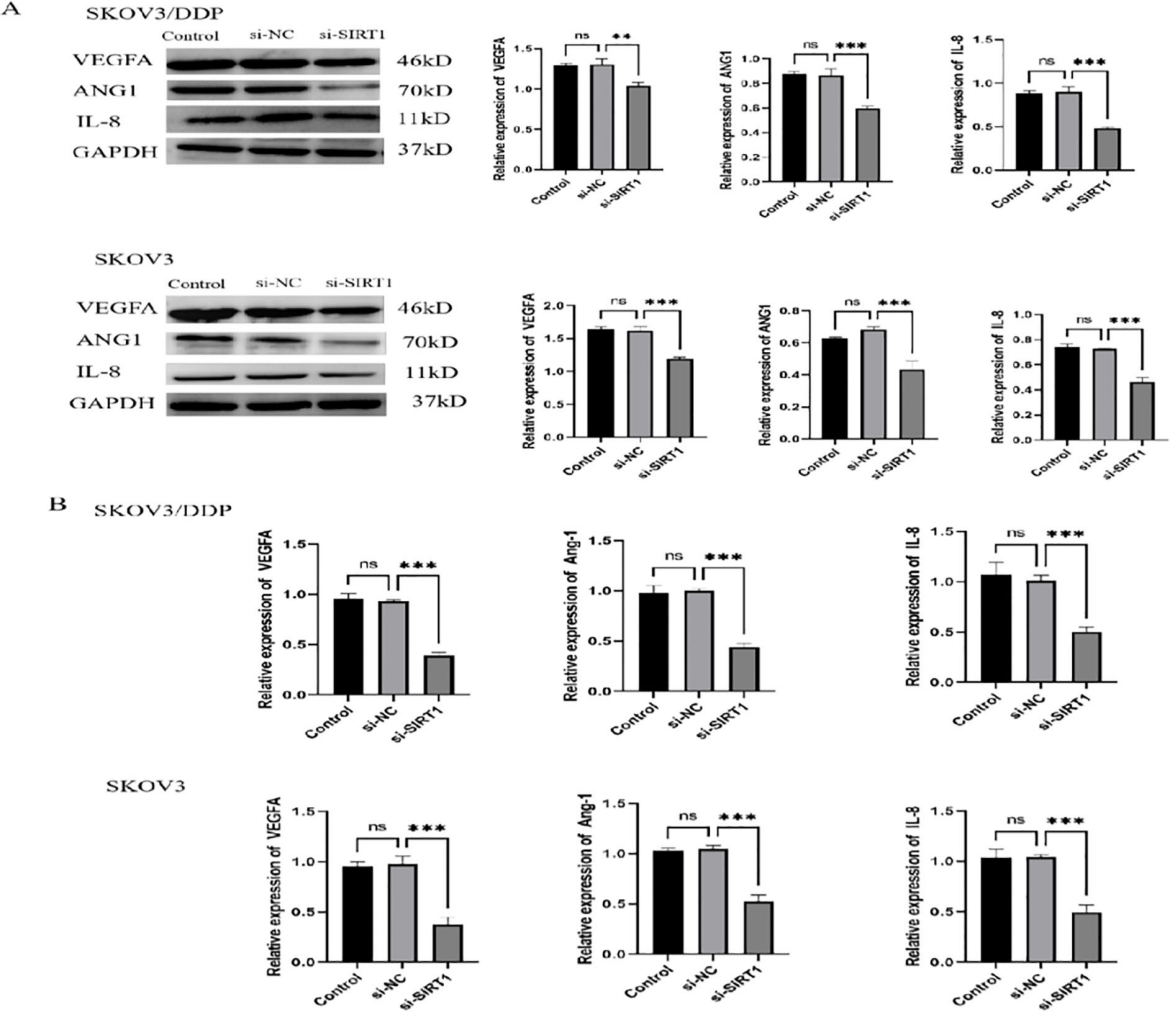
Suppression of angiogenesis in ovarian cancer cells by knocking down SIRT1. **(A)** The tube forming ability of cells in si-NC group and si-SIRT1 group was detected by tube forming experiment. **(B)** Western Blotting was used to detect the protein expression levels of angiogenic factors (VEGFA, Ang-1, IL-8) in si-NC group and si-SIRT1 group. **(C)** The mRNA expression levels of angiogenic factors (VEGFA, Ang-1, IL-8) in si-NC group and si-SIRT1 group were detected by RT-PCR. **P < 0.01, ***P < 0.001.

In order to study the effect of SIRT1 on glycolysis and angiogenesis of ovarian cancer cells, we detected the glucose uptake, lactic acid production, ATP level, ROS level and NADPH/NADP+ ratio of SKOV3 and SKOV3/DDP cells after SIRT1 was knocked down. The results showed that knocking down SIRT1 significantly reduced glucose uptake and lactic acid production of SKOV3 and SKOV3/DDP cells (**Figures 3A, B**), decreased ATP level (**Figure 3C**), increased ROS level (**Figure 3D**) and decreased NADPH/NADP+ ratio (**Figure 3E**) compared with si-NC group. The results of Western Blotting and qRT-PCR showed that knocking down SIRT1 significantly reduced the protein and mRNA expression levels of key glycolytic enzymes (HXK2, PKM, LDHA, PDHA1) and glucose metabolites (ALDOA, GLUT1) in SKOV3 and SKOV3/DDP cells compared with si-NC group (**Figures 3F, G**). In addition, the experimental results of tube formation showed that knocking down SIRT1 significantly inhibited the tube formation ability of SKOV3 and SKOV3/DDP cells compared with the si-NC group, and knocking down SIRT1 significantly decreased the protein and mRNA expression levels of angiogenic factors (VEGFA, Ang-1, IL-8) in SKOV3 and SKOV3/DDP cells compared with the si-NC group (**Figures 4A, B**).

### SIRT1 regulates glycolysis and angiogenesis of ovarian cancer cells through β-catenin/c-myc/PKM2 pathway

SIRT1 regulates glycolysis and angiogenesis of ovarian cancer cells through β-catenin/c-myc/PKM2 pathway.

**Figure 5:**
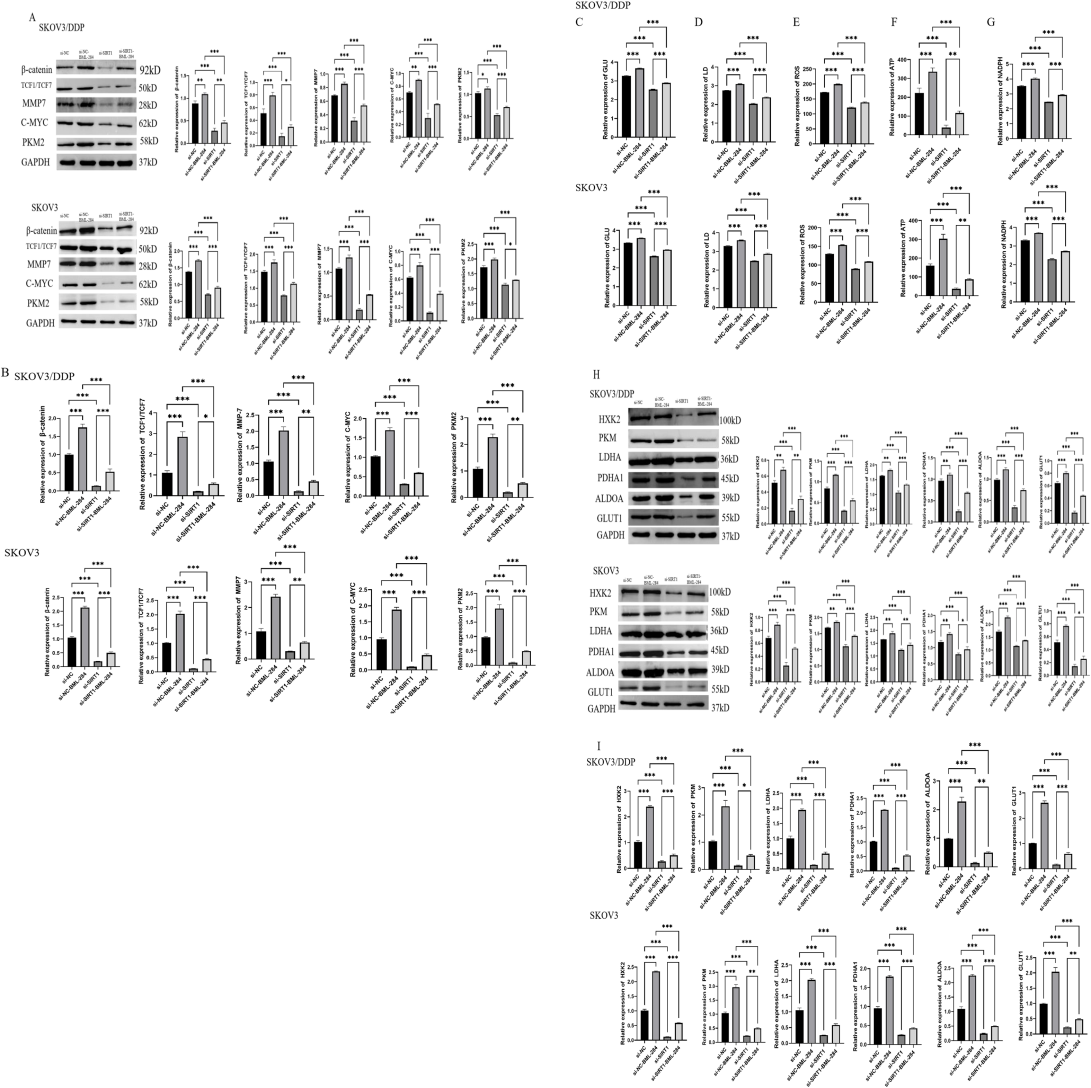
SIRT1 regulates glycolysis of ovarian cancer cells through β-catenin/c-myc/PKM2 pathway. **(A)** Western Blotting was used to detect the protein expression levels of β-catenin, c-myc and PKM2 in cells of si-NC group, si-NC+BML-284 group, si-SIRT1 group and si-SIRT1+BML-284 group. **(B)** PCR was used to detect the protein expression levels of β-catenin, c-myc and PKM2 in si-NC group, si-NC+BML-284 group, si-SIRT1 group and si-SIRT1+BML-284 group. **(C)** Glucose uptake test was used to detect the glucose uptake capacity of cells in si-NC group, si-NC+BML-284 group, si-SIRT1 group and si-SIRT1+BML-284 group. **(D)** The lactic acid production of cells in si-NC group, si-NC+BML-284 group, si-SIRT1 group and si-SIRT1+BML-284 group was detected by lactic acid determination experiment. **(E)** ATP level of cells in si-NC group, si-NC+BML-284 group, si-SIRT1 group and si-SIRT1+BML-284 group was detected by ATP determination experiment. **(F)** The ROS levels of cells in si-NC group, si-NC+BML-284 group, si-SIRT1 group and si-SIRT1+BML-284 group were detected by ROS assay. **(G)** NADPH/NADP+ assay The NADPH/NADP+ ratios of cells in si-NC group, si-NC+BML-284 group, si-SIRT1 group and si-SIRT1+BML-284 group were detected. **(H)** Western Blotting was used to detect the protein expression levels of key glycolytic enzymes (HK2, PKM2, LDHA, GLUT1) in si-NC group, si-NC+BML-284 group, si-SIRT1 group and si-SIRT1+BML-284 group. **(I)** PCR was used to detect the protein expression levels of key glycolytic enzymes (HK2, PKM2, LDHA, GLUT1) in si-NC group, si-NC+BML-284 group, si-SIRT1 group and si-SIRT1+BML-284 group. *P < 0.05, **P < 0.01, ***P < 0.001.

**Figure 6:**
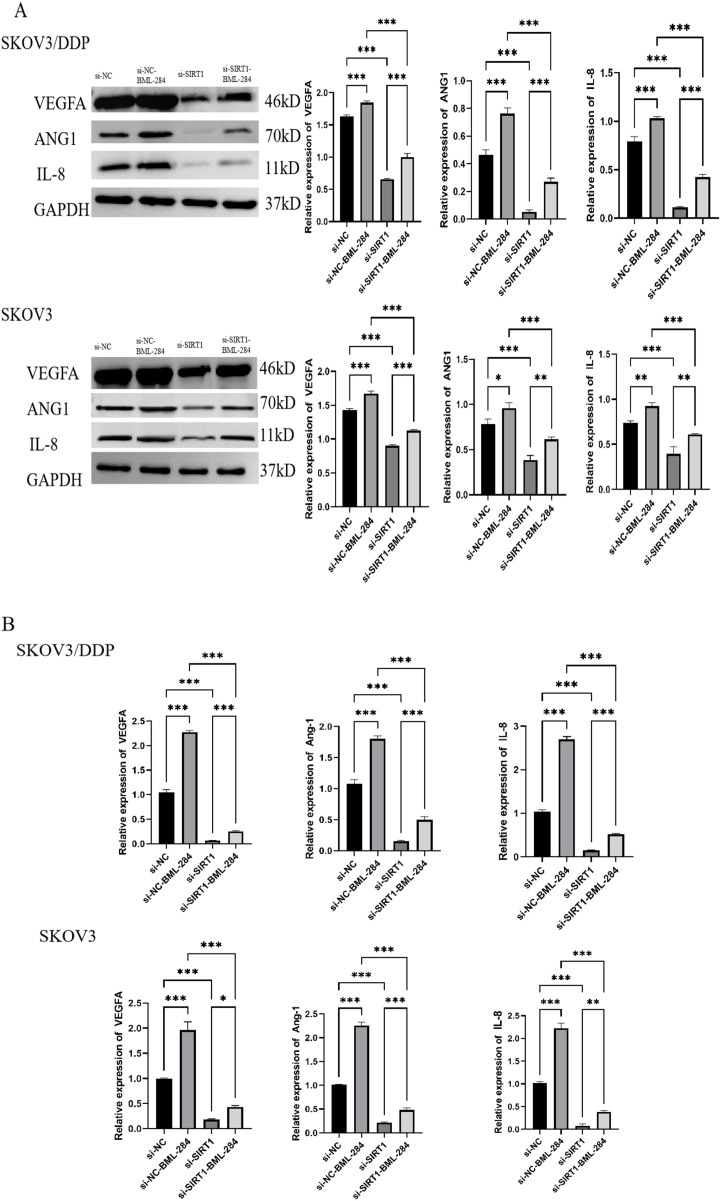
SIRT1 regulates angiogenesis of ovarian cancer cells through β-catenin/c-myc/PKM2 pathway. **(A)** Western Blotting was used to detect the protein expression levels of angiogenesis factors (VEGFA, Ang-1, IL-8) in si-NC group, si-NC+BML-284 group, si-SIRT1 group and si-SIRT1+BML-284 group. **(B)** The mRNA expression levels of angiogenic factors (VEGFA, Ang-1, IL-8) in si-NC group, si-NC+BML-284 group, si-SIRT1 group and si-SIRT1+BML-284 group were detected by QRT-PCR. *P < 0.05, **P < 0.01, ***P < 0.001.

In order to study whether SIRT1 regulates glycolysis and angiogenesis of ovarian cancer cells through β-catenin/c-myc/PKM2 pathway, we used β-catenin agonist BML-284 to intervene. Western Blotting and PCR results showed that knocking down SIRT1 significantly decreased the protein expression levels of β-catenin, TCF1/TCF7, MMP-7, c-myc and PKM2 in SKOV3 and SKOV3/DDP cells compared with si-NC group. However, BML-284 can reverse the inhibition of SIRT1 on the expression of β-catenin, TCF1/TCF7, MMP-7, c-myc and PKM2 (**Figures 5A, B**). Further experimental results show that BML-284 can reverse the inhibition of SIRT1 on glucose uptake, lactic acid production, ATP level, ROS level, NADPH/NADP+ ratio, and the expression of key glycolytic enzymes and angiogenic factors (**Figures 5C–I**, **6A–C**). This suggests that SIRT1 regulates glycolysis and angiogenesis of ovarian cancer cells through β-catenin/c-myc/PKM2 pathway.

### Knocking down SIRT1 can inhibit the damage of tumor to liver and kidney function

**Figure 7:**
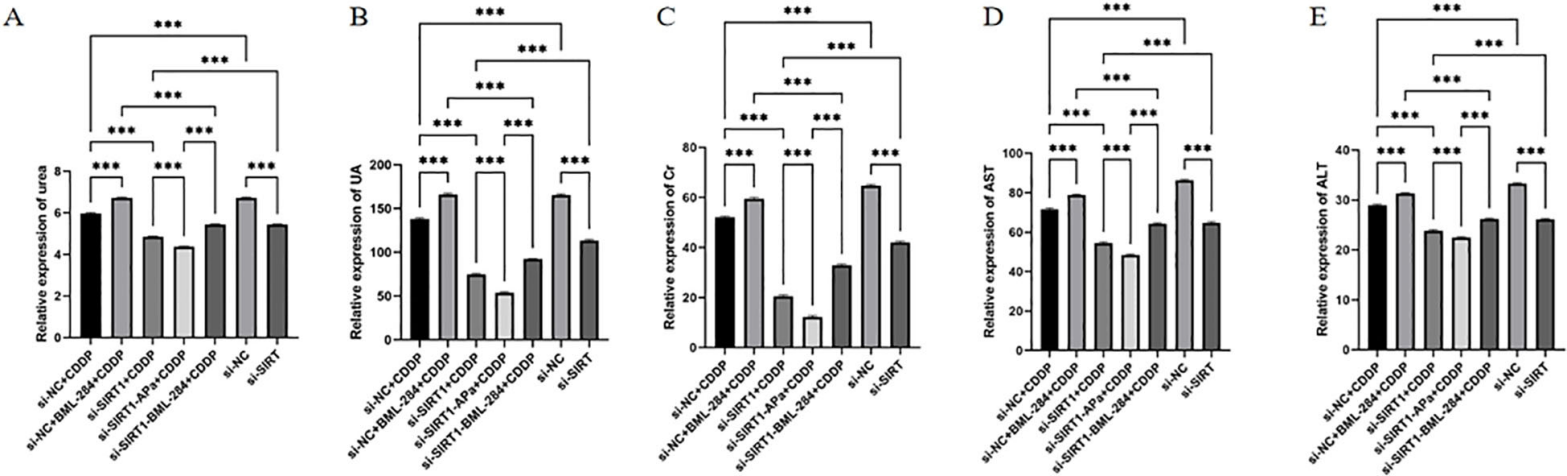
Knocking down SIRT1 inhibits the damage of ovarian cancer to liver and kidney function. **(A-C)** Release level of renal function (Cr, Urea and UA). **(D, E)** Release level of liver function (ALT and AST). ***P < 0.001.

In order to verify the effect of SIRT1 *in vivo*, we constructed a nude mouse model of transplanted tumor. In animal experiments, in order to evaluate the *in vivo* efficacy and potential toxicity of SIRT1 knock-down combined with or without BML-284 and apatinib on cisplatin-resistant ovarian cancer, we first detected the related indexes of liver and kidney function in each group of mice. The results of ELISA (**Figure 7**) showed that compared with the Si-NC group, the levels of ALT, AST, Cr, Urea and UA in the Si-SIRT1 group decreased significantly, indicating that knocking down SIRT1 alone can inhibit the damage of liver and kidney function in ovarian cancer. After cisplatin (CDDP) treatment, the levels of ALT, AST, Cr, Urea and UA were inhibited to some extent, but the anti-tumor effect of SIRT1 knockdown combined with cisplatin (si-SIRT1+CDDP) was significantly better than that of cisplatin alone (si-NC+CDDP), which indicated that SIRT1 knockdown enhanced the therapeutic effect of cisplatin. Apatinib combined with SIRT1 knockdown and cisplatin (si-SIRT1+APa+CDDP) showed the strongest inhibitory effect on ALT, AST, Cr, Urea and UA levels. On the contrary, activating β-catenin pathway (si-NC+BML-284+CDDP) slightly promoted the release of ALT, AST, Cr, Urea and UA, and activating β -catenin (si-SIRT1+BML-284+CDDP) on the basis of SIRT1 knockdown combined with cisplatin partially reversed SIRT1 knockdown (Si-SIR). This suggests that SIRT1 knockdown combined with apatinib and cisplatin does not cause significant hepatorenal toxicity in the treatment of tumors, suggesting that the combined treatment scheme is safe.

### SIRT1 promotes glycolysis and angiogenesis of ovarian cancer by regulating β-catenin/c-myc/PKM2 pathway

**Figure 8:**
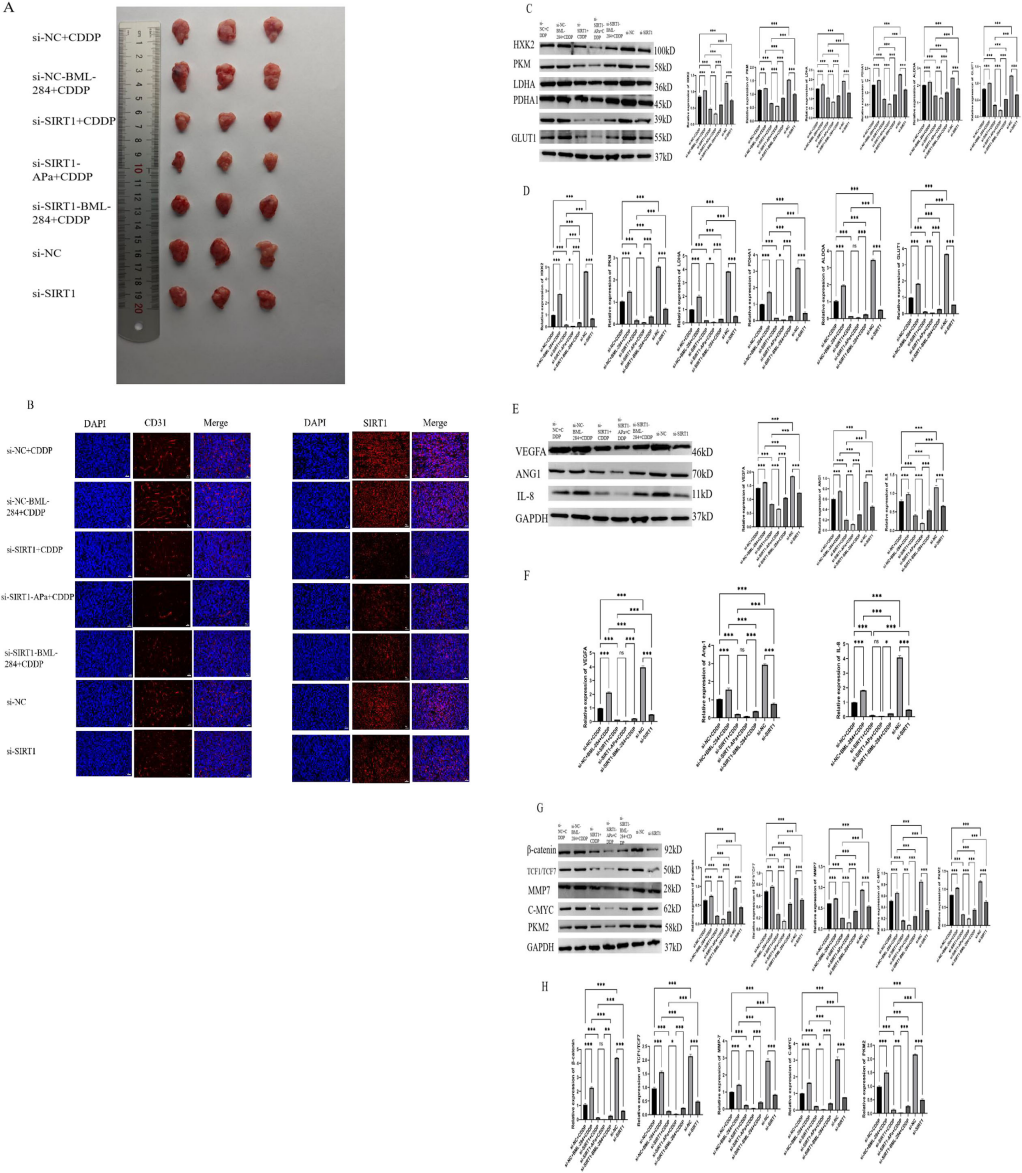
SIRT1 promotes glycolysis and angiogenesis of ovarian cancer by regulating β-catenin/c-myc/PKM2 pathway. **(A)** tumor volume. **(B)** Immunofluorescence staining was used to detect the expression of SIRT1 and CD31 in tumor tissues. **(C, D)** Western Blotting and PCR were used to detect the protein expression levels of key glycolytic enzymes (HK2, PKM2, LDHA and GLUT1, etc.) in tumor tissues. **(E, F)** Western Blotting and PCR were used to detect the protein expression levels of angiogenesis factors (VEGFA, Ang-1, IL-8) in tumor tissues. **(G, H)** Western Blotting and PCR were used to detect the mRNA expression levels of β-catenin/c-myc/PKM2 pathway related factors (β-catenin, TCF1/TCF7, MMP-7, C-myc and PKM2) in tumor tissues. *P < 0.05, **P < 0.01, ***P < 0.001.

In order to verify the effect of SIRT1 on β-catenin/c-myc/PKM2 pathway, we used WB and PCR to detect related proteins. The experimental results showed that compared with si-NC, si-SIRT1 significantly inhibited the growth of ovarian cancer cells *in vivo* (**Figure 8A**). The results of immunofluorescence staining showed that knocking down SIRT1 significantly reduced the expression of SIRT1 and CD31 in tumor tissues compared with si-NC group (**Figure 8B**). Western Blotting and qRT-PCR results showed that compared with si-NC, si-SIRT1 reduced the expression levels of angiogenesis-related proteins (VEGFA, Ang-1, IL-8) and glycolytic key proteins (HXK2, PKM2, LDHA, etc.) in tumor tissues (**Figures 8C, D**), while inhibiting β-catenin/c-myc. Cisplatin (CDDP) treatment inhibited tumor growth to a certain extent, but the anti-tumor effect of SIRT1 knock-down combined with cisplatin (si-SIRT1+CDDP) was significantly better than that of cisplatin alone, indicating that SIRT1 knock-down enhanced the therapeutic effect of cisplatin. Furthermore, Apatinib combined with SIRT1 knockdown and cisplatin (si-SIRT1+APa+CDDP) showed the strongest tumor growth inhibition effect and the lowest levels of angiogenesis and glycolysis. On the contrary, activating β-catenin pathway (si-NC+BML-284) slightly promoted tumor growth, and activating β -catenin (Si-SIRT1+BML-284+CDDP) on the basis of SIRT1 knockdown combined with cisplatin partially reversed the antitumor effect of Sirt1 knockdown. This suggests that SIRT1 knockdown can inhibit tumor angiogenesis and glycolysis by inhibiting β-catenin/c-myc/PKM2 pathway, thus inhibiting the growth of ovarian cancer cells *in vivo* and enhancing the anti-tumor effect of cisplatin.

## Discussion

In this study, it was found that the expression of SIRT1 increased in ovarian cancer cells, especially in cisplatin-resistant cell line SKOV3/DDP. Knocking down SIRT1 can inhibit the proliferation, invasion and migration of ovarian cancer cells, promote cell apoptosis, and reduce the drug resistance of cells to cisplatin. The mechanism study shows that SIRT1 can promote the glycolysis and angiogenesis of ovarian cancer cells by activating β-catenin/c-myc/PKM2 pathway and up-regulating the expression of key enzymes of glycolysis and angiogenesis factors. *In vivo* experiments, knocking down SIRT1 can inhibit tumor growth and reduce the glycolysis level and angiogenesis ability of tumor tissue.

SIRT1 is a highly conserved nicotinamide adenine dinucleotide (NAD)-dependent deacetylase, which is mainly located in the nucleus and mitochondria. SIRT1 plays crucial roles in maintaining cellular homeostasis under physiological conditions. It regulates a wide range of biological processes, including energy metabolism, stress response, genomic stability, autophagy, and aging. Through deacetylation of histones and non-histone proteins such as p53, FOXOs, and PGC-1α, SIRT1 modulates gene expression and signaling pathways involved in oxidative stress resistance, mitochondrial biogenesis, and apoptosis. In normal cells, SIRT1 acts as a metabolic sensor that links cellular energy status to adaptive responses, thereby promoting survival under nutrient deprivation or oxidative stress. However, in cancer contexts including ovarian cancer, SIRT1’s normal regulatory functions are often hijacked to support tumor progression, metabolic reprogramming, and therapy resistance, highlighting its dual role as both a protector in normal physiology and a promoter in pathology. Silencing information regulator 2 was discovered in the study of yeast transcription silencing, and then Frye et al. (18)found that there are five homologous genes in human body, abbreviated as SIRT1~5. SIRT1 can participate in a series of cell metabolism and function regulation by modifying histones and non-histones to change some targets (19). In recent years, it has been found that the expression of SIRT1 is abnormal in many tumors, which is closely related to the occurrence and development of tumors (20). In ovarian cancer, the expression of SIRT1 is usually increased, which is associated with poor prognosis (8). The results of this study are consistent with previous studies, which further confirmed the role of SIRT1 in promoting ovarian cancer.

Glycolysis not only provides energy for tumor cells, but also provides intermediate metabolites for cell growth and proliferation (21, 22). For example, the key enzymes in glycolytic pathway, such as hexokinase 2 (HK2), pyruvate kinase M2 (PKM2) and lactate dehydrogenase A (LDHA), are highly expressed in many tumors and are closely related to the malignant biological behavior of tumors (22–24). Angiogenesis is a necessary condition for tumor growth and metastasis. Tumor cells secrete angiogenic factors, such as vascular endothelial growth factor A (VEGFA), angiopoietin-1 (Ang-1) and interleukin-8 (IL-8) (5). The abnormal proliferation of tumor blood vessels not only provides a way for tumor cells to survive and spread, but also provides microenvironment support for tumor chemotherapy resistance (25). This study found that SIRT1 can promote glycolysis and angiogenesis of ovarian cancer cells, thus enhancing the malignant biological behavior of tumors. Specifically, knocking down SIRT1 can significantly reduce glucose uptake, lactic acid production and ATP levels of ovarian cancer cells, while increasing ROS levels and reducing NADPH/NADP+ ratio. These results indicate that the inhibition of SIRT1 can effectively interfere with the energy metabolism of ovarian cancer cells, making them unable to meet the demand of rapid proliferation. In addition, knocking down SIRT1 can also significantly inhibit the angiogenesis ability of ovarian cancer cells and reduce the expression of angiogenic factors such as VEGFA, Ang-1 and IL-8. These results indicate that the inhibition of SIRT1 can effectively inhibit the angiogenesis of ovarian cancer cells, thus blocking the nutritional supply and metastasis of tumors.

Wnt/β-catenin signaling pathway plays an important role in embryonic development, tissue homeostasis and tumorigenesis (26, 27). β-catenin is a key molecule in Wnt signaling pathway. Its accumulation and nucleation in cytoplasm can activate the transcription of downstream target genes and promote cell proliferation and survival. C-myc is an important transcription factor, which is involved in the regulation of cell proliferation, differentiation and apoptosis (28). PKM2 is a key enzyme in glycolytic pathway, and its increased expression can promote glycolysis of tumor cells (29). Studies have shown that β-catenin, c-myc and PKM2 play an important role in glycolysis, angiogenesis and drug resistance of tumors (29–31). This study found that knocking down SIRT1 can significantly reduce the protein expression levels of β-catenin, c-myc and PKM2 in ovarian cancer cells. BML-284, a β-catenin agonist, can reverse the inhibitory effect of SIRT1 on the expression of β-catenin, c-myc and PKM2 proteins, and restore the glycolysis and angiogenesis of ovarian cancer cells. These results indicate that SIRT1 regulates glycolysis and angiogenesis of ovarian cancer cells by activating β-catenin/c-myc/PKM2 pathway.

In order to verify the effect of SIRT1 *in vivo*, we constructed a nude mouse model of transplanted tumor. First, it was verified that knocking down SIRT1 can inhibit the damage of tumor to liver and kidney function, and it was also found that knocking down SIRT1 can inhibit the growth of ovarian cancer cells *in vivo*. SIRT1 knockdown significantly reduces the level of angiogenesis and glycolysis in tumor tissues by inhibiting β-catenin/c-myc/PKM2 pathway, thus inhibiting tumor growth, which is consistent with the effect of SIRT1 on cell proliferation, migration and metabolism observed *in vitro*. More importantly, SIRT1 knockdown enhanced the antitumor effect of cisplatin, suggesting that SIRT1 may become a new target to overcome chemotherapy resistance of ovarian cancer. The synergistic effect of apatinib combined with SIRT1 knockdown and cisplatin further verified the potential of targeted angiogenesis and metabolic pathway in the treatment of ovarian cancer.

To sum up, this study shows that the expression of SIRT1 is increased in ovarian cancer cells, which can promote glycolysis and angiogenesis of ovarian cancer cells by activating β-catenin/c-myc/PKM2 pathway, thus enhancing chemotherapy resistance. SIRT1 is expected to be a new target for ovarian cancer treatment. At present, some SIRT1 inhibitors have entered the clinical trial stage to treat various diseases, including tumors. However, the specificity and safety of these SIRT1 inhibitors still need further evaluation. In addition, the combined treatment strategy for SIRT1, such as the combination of SIRT1 inhibitors with chemotherapy drugs, angiogenesis inhibitors or immune checkpoint inhibitors, is also worthy of further exploration. However, this study also has limitations. First, our conclusions are primarily derived from SKOV3 and its resistant variant SKOV3/DDP cell lines; validation in additional ovarian cancer models is warranted. Second, while our *in vivo* data suggest that the combination of SIRT1 inhibition with apatinib and cisplatin was well-tolerated and effective, the assessment of hepatorenal toxicity was observational and secondary to tumor response. A comprehensive toxicological evaluation in appropriate models is necessary before any clinical translation. Future studies should focus on elucidating the detailed pharmacokinetic and safety profiles of SIRT1-targeted agents in combination with existing therapies, and explore their efficacy in patient-derived xenografts or clinical settings.

## Data Availability

The raw data supporting the conclusions of this article will be made available by the authors, without undue reservation.
